# The Insulin-Like Growth Factor System and Nutritional Assessment

**DOI:** 10.6064/2012/768731

**Published:** 2012-07-08

**Authors:** Callum Livingstone

**Affiliations:** ^1^Peptide Hormones Supraregional Assay Service (SAS), Clinical Biochemistry Department, Royal Surrey County Hospital, Guildford, Surrey GU2 7XX, UK; ^2^Faculty of Health and Medical Sciences, University of Surrey, Guildford, Surrey GU2 5XH, UK

## Abstract

Over recent years there has been considerable interest in the role of the insulin-like growth factor (IGF) system in health and disease. It has long been known to be dysregulated in states of under- and overnutrition, serum IGF-I levels falling in malnourished patients and responding promptly to nutritional support. More recently, other proteins in this system have been observed to be dysregulated in both malnutrition and obesity. Currently no biochemical marker is sufficiently specific for use in screening for malnutrition, but levels may be valuable in providing information on nutritional status and in monitoring of nutritional support. All have limitations as nutritional markers in that their serum levels are influenced by factors other than nutritional status, most importantly the acute phase response (APR). Levels should be interpreted along with clinical findings and the results of other investigations such as C-reactive protein (CRP). This paper reviews data supporting the use of proteins of the IGF system as nutritional markers.

## 1. Introduction

The insulin-like growth factor (IGF) system consists of the peptide hormones IGF-I and -II, their cell surface receptors, and IGF-binding proteins (IGFBPs). IGF-I and -II are the principal components of the system. They have biological roles in mediating the effects of growth hormone (GH) and during development [1]. Their actions can be divided into rapid metabolic effects and longer-term growth-promoting effects [2]. Compared to insulin both circulate in relatively high concentrations in serum. They are also released into interstitial fluids where they act locally in a paracrine or autocrine manner. The IGFs generate their biological responses by activating a receptor tyrosine kinase (RTK) which initiates an intracellular signalling cascade [3]. Their function is modulated by the IGFBPs [4].

### 1.1. The IGFs and Their Receptors

IGF-I exerts acute anabolic actions on protein and carbohydrate metabolism by increasing the cellular uptake of amino acids. As such, it is an important regulator of muscle mass. It has long-term effects on cell proliferation, differentiation, and apoptosis [4, 5]. It is a potent mitogen increasing DNA synthesis and accelerating cell cycle progression. IGF-I is predominantly synthesised in liver, in response to GH, but is also synthesised to a lesser extent in various other tissues. Other factors which regulate its production include genetic factors, nutrition, and other hormones such as thyroxine, gonadal steroids, and cortisol. Its biological half-life in serum is only a few hours. Most serum IGF-I is bound to IGFBP-3 and other proteins of this family. A small proportion (<1%) which exists as free IGF-I is thought to be the bioactive component. Serum IGF-I levels are low at birth and increase through puberty to the age of 20 years, thereafter declining gradually with age [6]. Levels must therefore be interpreted using age-related reference ranges. Disease-related dysregulation of IGF-I may affect local or systemic actions of IGF-I. Localised growth disturbance may occur, for example, in bone disease where the pathology is confined to a specific region [7–9]. Disturbance of systemic IGF-I actions is implicated in metabolic disorders such as diabetes, malnutrition, obesity, acromegaly, and GH deficiency. Its measurement in serum has an established place in the clinical management of the latter two conditions.

IGF-II is a polypeptide synthesised mainly in liver which, in its mature form, is about 70% identical to IGF-I [10]. In comparison to IGF-I, its physiological role and regulation are relatively poorly understood but it is thought to be involved in foetal differentiation, metabolic regulation, cell growth, and cell division, being a more potent mitogen than IGF-I. The principal regulator of IGF-II gene transcription is unclear, GH having no effect on its expression in humans, but its expression is influenced by various other hormones and growth factors [5, 11]. In serum, IGF-II occurs at levels around three times higher than those of IGF-I. The concentration of biologically active free IGF-II parallels that of total IGF-II. IGF-II has been observed to be dysregulated in a variety of disease states notably malnutrition, obesity, and non-islet cell tumour hypoglycaemia (NICTH), a rare condition in which tumours secrete excessive IGF-II [12–14]. IGF-II is less often measured than IGF-I in clinical contexts, the only recognised indication for its measurement being investigation of suspected NICTH.

The IGFs bind principally to two cell surface transmembrane receptors, namely, the IGF receptor type 1 (IGF-1R) and IGF receptor type 2 (IGF-2R), but they also bind to the insulin receptor (I-R) [15]. The acute and long-term biological actions of the IGFs are mediated via binding to the IGF-1R and activation of its RTK activity [3, 16]. The IGF-2R has no signalling activity. It is thought to regulate IGF-II availability to receptors by sequestering it and enabling its degradation [10].

### 1.2. IGF Binding Proteins (IGFBPs)

The IGFBPs are a family of six proteins which bind the IGFs thereby regulating their availability to receptors. They are synthesised in liver, connective tissue, and various other cell types [7]. The individual proteins have distinct binding preferences, IGFBP-2, for example, binding preferentially to IGF-II [17]. The functions of the IGFBPs *in vivo* are only partly understood, but suggested functions and other features of the IGFBPs are listed in Table 1.

In circulation they are present predominantly as 150 kDa ternary complexes consisting of IGF-I or -II, IGFBP-3, and acid-labile subunit (ALS), a protein synthesised mainly in liver [11]. The remainder of circulating IGFs are present mainly as binary complexes with IGFBPs or in the free form [2]. Ternary complexes are unable to cross endothelia and so extend the half-life of IGFs in the circulation. Various mechanisms regulate release of IGFs from these complexes allowing interaction with receptors [18]. Proteases have been identified which can fragment IGFBPs enhancing IGF release [7]. A variety of catabolic states are known to be accompanied by increased proteolysis of IGFBP-3. IGFBPs are also degraded by matrix metalloproteinases. Release of the IGFs from their binding proteins is also regulated by IGFBP phosphorylation and cell surface association [19].

## 2. Malnutrition in Hospitalised Patients

Malnutrition is common amongst hospital patients, in whom it predisposes to disease, increases the risk of complications, and delays recovery. As well as being associated with an adverse outcome, these factors tend to increase the length of stay (LOS) in hospitals which has considerable cost implications. It is therefore vital to detect malnutrition as early as possible in order that nutritional support can be commenced. Nutritional support buys time for therapeutic interventions to aid recovery and reduces morbidity and mortality [20]. It can be delivered orally, enterally, or parenterally depending on the clinical situation. The first two routes are the more physiological, parenteral nutritional (PN) being reserved for patients with intestinal failure in whom the gut is either dysfunctional or inaccessible.

It is well recognised that malnutrition in hospitals is often overlooked and consequently undertreated. Nutritional screening is therefore widely advocated by professional bodies, so that patients at risk of malnutrition can be identified [21]. Whilst a number biochemical tests are sensitive for the detection of malnutrition, their diagnostic specificity is too low for use in screening. Currently, nutritional screening is carried out using screening tools such as the malnutrition universal screening tool (MUST) [22].

## 3. Nutritional Assessment of Patients

Once the decision has been taken to provide nutritional support for an individual, it is essential that their nutritional requirements are met if the full benefit is to be realised. When patients are referred for nutritional support, the dietitian should carry out a formal nutritional assessment, part of which should include an estimation of the patient's energy and protein requirements. Energy requirements can be calculated based on body weight and age using a method such as the Schofield equation to determine the approximate basal metabolic rate (BMR) [23, 24]. Protein requirements can be calculated from body weight. These methods only provide estimates which can be significantly altered in, for example, critical illness, and it should be borne in mind that the final arbiters of the success of nutritional support are weight restoration and recovery. Following commencement of nutritional support, individual patients must therefore be closely monitored for evidence of nutritional repletion, irrespective of whether estimated requirements have been met. Where the patient is not recovering, consideration needs to be given to adjust the nutritional support provision. The National Institute for Health and Clinical Excellence (NICE) guidelines for nutritional support in adults (2006) provide guidance on the monitoring of patients [25]. Both anthropometric measurements and the results of biochemical tests contribute to this process.

## 4. Protein Markers in Nutritional Assessment

Biochemical markers can be useful adjuncts in the nutritional assessment and monitoring nutritional support, though their clinical utility is currently a topic of much debate and research. Markers currently available include transferrin, prealbumin, and retinol-binding protein, the serum levels of which may be subnormal in malnutrition. The current NICE guidelines do not advocate measurement of these as part of the laboratory monitoring of nutritional support [25], but their levels have been widely measured in research studies investigating malnutrition. Novel biochemical markers are likely, in time, to find a place in clinical management of patients. However, the assessment of novel nutritional markers is complicated in that there is no universally accepted “gold standard” method for nutritional assessment, making it difficult to define the diagnostic performance of new markers. Instead, studies have compared novel markers to methods such as detailed nutritional assessment and subjective global assessment [26].

### 4.1. Limitations of Protein Markers

All nutritional marker proteins in current use suffer from the limitation that factors other than nutritional status influence their serum concentrations, for instance, hydration status, disease, surgery, and abnormal liver function [27]. In practice it is difficult to separate the influence of these factors from that of malnutrition. The most problematic confounder is the acute phase response (APR) which causes decreased levels of these proteins [28]. During the APR, cytokines such as interleukin-6 (IL-6) precipitate a decline in the protein concentrations over a characteristic time course [29]. They do this by increasing capillary permeability, increasing protein catabolism, and reducing the hepatic synthesis of proteins [30]. The magnitude of the reduction in levels correlates with the severity of the injury [31, 32]. These changes occur independently of total body protein status and irrespective of whether the patient is receiving adequate nutritional support [29]. Levels of nutritional marker proteins tend to be inversely related to the levels of APR markers such as C-reactive protein (CRP). Measurement of CRP is therefore of value in interpretation. The APR is particularly problematic in hospitalised patients where most patients in need of nutritional support are also acutely ill. Nutritional markers may therefore find greater use in outpatients or in the context of epidemiological research where subjects are relatively well. Their responsiveness to changes in nutritional status may make them of more value in monitoring than in baseline assessment of the patient.

Although the available markers may be poor at detecting nutritional status during acute illness, they are recognised to have prognostic value and as such may be useful parameters to include in a biochemical profile in patients referred for nutritional support. This prognostic value relates to their place as negative APR markers. It is worthwhile to consider the characteristics of the ideal nutritional marker which would not be subject to any of the limitations discussed (Table 2). Whilst the ideal marker does not exist, it is useful to keep this list in mind when considering the merits of current or novel markers. The remainder of this paper focuses on proteins of the IGF axis as potential markers of nutritional status.

## 5. The Role of IGF Axis Proteins in Managing Malnutrition and Nutritional Assessment

Serum levels of proteins of the IGF system have been widely reported to be altered in malnutrition. In view of this, there is increasing interest in their potential clinical utility in nutritional assessment of patients and in monitoring nutritional support provision.  Table 3 summarises the effects of malnutrition and obesity on levels of the IGFs and IGFBPs. To date IGF-I is the most extensively researched member of this family [33–35].

### 5.1. IGF-I

Currently, the main clinical use of IGF-I measurement is in the assessment of pituitary GH status, that is, to detect deficiency in the case of hypopituitarism and excess in acromegaly. However, its serum level has also long been recognised to reflect nutritional status, declining during fasting and starvation [46, 61]. Following one week of starvation, levels are comparable to those observed in hypopituitarism. It correlates with anthropometric indices such as BMI and the levels of other protein nutritional marker proteins [62]. The decline in its levels appears to be greater in those with protein energy malnutrition as compared to protein malnutrition alone [63]. The precise mechanism for this decline is not known but likely reflects the decrease in hepatic protein synthesis which accompanies malnutrition. Its physiological purpose may be to divert substrates away from growth and towards metabolic homeostasis, a process assisted by the relatively insulin-resistant state which exists in starvation. It may also contribute to the catabolic reaction observed in starvation and sepsis.

Conversely, IGF-I levels return to normal in response to nutritional support during which they have been observed to climb incrementally in relation to weight gain [46, 64, 65]. The short serum half life of IGF-I renders it more sensitive than previous markers to short-term changes in nutritional status, an attribute which increases its value in monitoring [66]. Moreover, serum IGF-I appears to be sensitive to both the amount and type of fat provided in nutritional support. Fish oil and low-fat solutions were significantly correlated to serum IGF-I, appearing to promote a more rapid recovery of its levels [67]. As well as being used as a marker of repletion, monitoring the IGF-I level may help to avoid overfeeding of patients and the adverse consequences thereof. Although it may have a place in monitoring, IGF-I like the older nutritional markers performs poorly as a screening test for malnutrition [30]. IGF-I is considered in more detail below in various clinical situations associated with malnutrition.

#### 5.1.1. Anorexia Nervosa

Malnourished patients with anorexia nervosa (AN) often have multiple endocrine abnormalities, including amenorrhoea, hypothyroidism, hypercortisolism, and elevated GH, with suppressed IGF-I reflecting GH resistance [34]. These abnormalities are believed to be secondary to nutritional deprivation as they also occur in starvation states secondary to other causes and return to normal upon weight restoration [68]. In patients with eating disorders, it has been demonstrated that serum IGF-I levels are closely related to BMI, body fat, and body muscle mass, reflecting the severity of nutritional depletion [36]. When treating AN, the aim is nutritional rehabilitation. Given that the serum IGF-I level responds to nutritional support, it has been investigated as an indicator of nutrition during treatment of adolescents with eating disorders [37]. Where there was net weight gain during treatment, IGF-I increased in parallel with the BMI standard deviation score, a measure of leanness. The authors concluded that IGF-I can be considered an indicator of nutritional status in these patients. It is sensitive to short-term weight changes and can be used at assessment and to monitor nutritional rehabilitation.

Assays are now available for free IGF-I enabling it to be measured in clinical studies. In common with total IGF-I, its levels fall in undernutrition [50, 51]. Free IGF-I has been reported to be more sensitive to short-term nutritional status than total IGF-I [69]. It is usually estimated by measurement of immunoreactivity of IGF-I in serum following extraction of IGFBPs. Although this method is valid in many cases, there are conditions such as chronic renal disease, obesity, and during fasting in which directly measured IGF-I bioactivity may more closely reflect the endogenous IGF-I bioactivity. A study was carried out in which IGF-I bioactivity was measured by assaying IGF-1R RTK activation. This was compared to total IGF-I and free IGF-I. IGF-I bioactivity was significantly reduced in patients with AN along with the two other parameters [1], all three parameters being closely correlated. This suggests that total IGF-I may considered as a surrogate marker for the IGF-I bioactivity. Whether these findings apply to malnourished states other than AN remains to be established. Studies of patients with AN have shown no change in IGFBP-3 proteolytic activity [50, 51]. More research needs to be carried out into the utility of free IGF-I as a nutritional marker.

#### 5.1.2. Paediatrics

Numerous studies have investigated the utility of IGF system proteins as nutritional markers in children. In malnourished children, serum IGF-I correlates strongly with height SD score suggesting that it is a useful indicator of growth and nutritional status [57]. It has also been reported to correlate with economic status independently of overt malnutrition [70].

Studies have investigated IGF-I as a marker of lean body mass (LBM) in malnourished children with various medical disorders. In cystic fibrosis (CF), the serum IGF-I level correlated with LBM, as evaluated by DEXA scanning, independently of weight [71]. The significance of this is that reduction in LBM can impair respiratory function thereby worsening the clinical outcome for these patients. IGF-I could therefore be used as be a tool for identifying patients with CF at risk of deterioration in respiratory function. In a separate study of children with CF, decreased IGF-I levels were shown to reflect growth retardation [58]. IGF-I was also used as a marker of LBM in a recent study of children commencing treatment for HIV infection [72]. During treatment, improved muscle mass, but not linear growth, was associated with normalised IGF-I concentrations. In children with congenital heart disease (CHD), the most important factor influencing serum IGF-I levels was the presence of cyanosis, levels being significantly lower in cyanotic compared to acyanotic patients [73]. The authors suggested that chronic hypoxia plays a significant role in the pathogenesis of malnutrition, reflected by serum IGF-I levels. Moreover, IGF-I deficiency in these patients may be responsible for the decrease in left ventricular mass.

Serum IGF-I has been used for monitoring children with short bowel syndrome (SBS) receiving PN, its level having been shown to improve in line with nitrogen balance [74]. It may therefore be of value along with other measurements in assessing protein nutritional status of these children. Not all children with SBS respond equally to standard nutritional approaches, and it is important to identify the subgroup who may respond poorly. IGF-I has been reported as an index of intestinal failure in children with SBS who merit more aggressive therapeutic intervention [75]. Intestinal transplantation can potentially liberate children with intestinal failure from the need for long-term PN, but it is difficult to achieve sufficient growth post-transplant in most patients. Negative linear growth velocity is predicted by low levels of IGF-I pre-transplant [76]. IGF-I levels may therefore be useful in identifying those patients requiring an intensive nutritional regimen after transplant. Recombinant growth hormone (rhGH) is another treatment that may reduce the amount of PN required in patients with SBS. This has recently been trialled in PN-dependent children [77]. Although they remained on PN, there was a significant increase in the amount of nutrition that could be delivered enterally rather than intravenously. This paralleled an increase in their energy balance over a period of 12 months following the treatment and serum IGF-I levels increased significantly.

#### 5.1.3. Critical Illness

Critical illness, in common with malnutrition, sepsis, and aging, is associated with a dramatic reduction in the mass and functional status of skeletal muscle which results from protein catabolism. This leads to muscle weakness and prolonged mechanical ventilation, factors which tend to increase LOS in ITU. There are a number of hormonal changes which occur in catabolic states including an increase in serum GH along with GH resistance leading to a reduction in both circulating IGF-I and its expression in skeletal muscle. There is also an increase in insulin resistance. In common with other protein markers, IGF-I is negatively influenced by inflammation, injury, and burns [28, 78–80]. Although the mechanism of this decrease is unclear, there is evidence that cytokines locally inhibit IGF-I expression, thereby contributing to catabolism [81].

In view of the role of IGF-I in influencing muscle mass and the pathophysiological role of its deficiency in critical illness, there has been interest in use of treatment with rhGH to increase IGF-I levels therapeutically. This treatment has been demonstrated to result in increased IGF-I levels with nitrogen retention and decreased LOS. However, two large randomised trials in 1999 reported increased mortality associated with infection and organ dysfunction [82]. Small clinical trials have reexamined rhGH supplementation in prolonged critical illness, demonstrating increased serum levels of IGF-I and IGFBP-3 in patients receiving adequate nutritional support. In a recent trial of 30 multiple trauma patients with prolonged critical illness, rhGH treatment delivered intravenously in pulses normalised IGF-I levels [83]. It is possible that rhGH supplementation may be safer and more efficacious in certain critically ill patients, but the subgroup likely to benefit needs to be defined. A clearer understanding of the GH resistance should lead to the development of new therapeutic strategies aimed at restoring the beneficial effects of anabolic agents such as GH and IGF-I. If in time the balance of evidence supports GH treatment in some critically ill patients, there may be a place for monitoring IGF-I levels. Current guidelines continue to recommend against therapeutic use of rhGH in critical illness [84].

#### 5.1.4. Obesity

Obesity is well recognised to influence the IGF system though the effects are relatively poorly understood [85]. An increase in BMI is associated with a reduction in GH secretion, but IGF-I does not appear to fall to the same extent. There is disagreement regarding the effect of obesity on IGF-I levels. Studies have reported IGF-I levels to be normal [38], high [39, 40], or low [41, 42]. Similarly there is variation between studies as to what happens to free IGF-I levels. These have been reported as normal [43], high [44, 45, 48, 49], or low [52], though the majority of studies suggest high levels. When IGF levels have been investigated in large populations, serum IGF-I levels appear to follow an inverted U-shaped association with BMI. Maximal IGF-I has been reported at BMI of 22–24 kg/m^2^ [48], 22.5–29.5 kg/m^2^, [60] and 30–35 kg/m^2^ [86] though the changes in IGF-I levels across the normal-to-high BMI range are relatively small. The elevation of free IGF-I in the obese at high BMI is a phenomenon that may be confined to nondiabetic subjects. In obese subjects with type 2 diabetes, free IGF-I was not significantly elevated when compared to lean controls [44]. Subjects with type 1 diabetes had a marked reduction (>50%) in free IGF-I [44]. More work is required to confirm these findings and to assess the mechanistic basis and consequences of overnutrition on free IGF-I levels.

Numerous studies have investigated how therapeutic interventions in obesity influence IGF-I levels. These are more consistent in showing a reduction in levels in response to a variety of approaches, namely, caloric restriction [45], gastric banding [87], physical activity [88], and a combination of dietary restriction and exercise training [89]. Whilst these findings are interesting, measurement of IGF-I in the context of obesity is currently confined to research studies. At present its measurement would not appear to be of value in guiding clinical management of obese patients. Allied to the study of IGF levels in obese subjects is an increasing interest in the link between obesity and cancer. It has been suggested that dysregulation of the IGF system plays a major role in this link [90]. Research will no doubt continue in this area, seeking to determine the underlying mechanisms, but it remains to be established whether measurement of IGF-I is of clinical value in predicting or monitoring mortality risk.

### 5.2. Other Proteins of the IGF System

#### 5.2.1. IGF-II

Relatively few studies have investigated IGF-II in nutritional contexts, but, in common with IGF-I, it has generally been observed to be dysregulated in both under- and overnutrition, its serum level falling in chronic malnutrition and climbing during refeeding [34, 46]. However, one study investigating short-term fasting observed no change in serum IGF-II [47]. IGF-II levels are reported to be elevated in obesity [49]. Following weight reduction in these subjects, both serum total and free IGF-II levels decreased significantly. More work is required to examine the physiological regulation of IGF-II and its dysregulation in disease. The utility, if any, of its measurement in nutritional contexts remains to be established.

#### 5.2.2. IGFBP-1

IGFBP-1 is expressed predominantly by liver and is abundant in blood [91], its expression being regulated by insulin which inhibits transcription of the IGFBP-1 gene. Its mRNA has a half life of only 2 hours which results in the level of IGFBP-1 in blood varying reciprocally with insulin levels [92]. However, IGFBP-1 levels lag behind insulin levels reflecting the longer half life of IGFBP-1 compared to insulin. This means that measurement of IGFBP-1 levels can be used to indirectly assess insulin secretion [91]. These findings have led to interest in IGFBP-1 as a marker of insulin resistance. In support of its use in this context, serum IGFBP-1 levels have been shown to correlate with the gold standard method for assessing insulin resistance, namely, the hyperinsulinaemic, euglycaemic clamp [93]. Low fasting serum IGFBP-1 levels correlate with hyperinsulinaemia and a variety of other cardiovascular risk factors, including increased BMI, fasting hypertriglyceridaemia, and low HDL-cholesterol concentrations all of which are features of the metabolic syndrome [94]. Indeed, its serum level has been suggested as a marker of metabolic syndrome in adults [53] and to be predictive of the development of insulin resistance in children [54]. Moreover, its levels increase in response to lifestyle interventions indicative of the reversible nature of insulin resistance upon life-style change [95]. In a study comparing it with other simple indices of insulin resistance in normal glucose-tolerant subjects, IGFBP-1 was suggested to be the most reliable marker [96]. It has the advantage of being a relatively stable analyte compared to insulin, thus having the potential to assess insulin resistance by means of a single fasting blood test. It may therefore have clinical utility in the assessment of cardiovascular risk in patients with metabolic syndrome and in monitoring the effect of therapeutic interventions.

IGFBP-1 has been less extensively studied in the context of undernutrition, but levels have been observed to be markedly elevated in patients with AN [51]. This is to be expected since low insulin levels during prolonged fasting have a permissive effect on IGFBP-1 gene transcription. It may be that the increase in IGFBP-1 during fasting acts as a protective mechanism against hypoglycaemia by binding IGF-I and thereby preventing its action. IGFBP-1 levels have been observed to be elevated and predictive of growth failure in patients with chronic renal failure [97].

#### 5.2.3. IGFBP-2

Relatively few studies have investigated IGFBP-2 in the context of malnutrition, but it appears that its serum levels rise in undernutrition and fall following recovery [34, 59, 102]. When compared with other proteins of the IGF system in a study of patients with AN, it was reported to be the best predictor of BMI [102]. In a large population cross-sectional study, IGFBP-2 levels decreased steadily with increasing BMI; the principle dietary factor determining its levels believed to be dairy protein [103]. IGFBP-2 is also of interest in metabolic syndrome and obesity, in which its serum levels fall [55]. Indeed, in a large population study, the reduction in IGFBP-2 levels associated with adiposity was reported to be the largest effect of adiposity observed on the IGF system [86]. Moreover, it has been suggested as an independent predictor of insulin resistance in a study comparing it to the hyperinsulinaemic euglycaemic clamp [56]. Although less well studied than IGFBP-1 in this context, it is potentially a more useful marker as its levels do not fluctuate in an insulin-dependent manner. Aside from nutritional contexts, IGFBP-2 levels are known to climb in NICTH and in other situations where the free IGF-II level is expected to be high [100]. Investigation of NICTH is currently the only established indication for its measurement.

#### 5.2.4. IGFBP-3

IGFBP-3 is the most abundant member of this family of proteins in serum. Its concentration generally parallels that of IGF-I both physiologically and in other clinical conditions and it is commonly measured along with the latter in the assessment of poor growth in children [104]. In studies of malnourished patients in which IGF-I levels have been observed to fall, the IGFBP-3 level also falls and recovers upon refeeding [57–59]. There is a consistent relationship between serum IGFBP-3 concentration and caloric intake. One study suggested that it was a more sensitive marker of nutritional status than IGF-I [58]. As such, its levels have potential utility in monitoring the adequacy of nutritional support regimens. In obesity the serum IGFBP-3 level appears to climb being positively associated with central adiposity though, unlike in malnutrition, its level does not necessarily parallel that of IGF-I [60]. Derangement of thyroid status is a potential confounding factor in the interpretation of IGFBP-3 levels. Serum IGFBP-3 and IGF-I levels correlate positively with free thyroxine levels, likely because thyroid hormones have a role in regulating the synthesis of these proteins [57].

## 6. Clinical Utility of IGF-I and Other Markers

The attraction of biochemical markers to the user is their objectivity. As such, they can complement clinical nutritional assessment, which is largely subjective in nature. This has prompted researchers to try to identify novel markers. To date a considerable evidence base supports the utility of IGF-I as a nutritional marker, its short half life in serum rendering it a more sensitive indicator of nutritional depletion than earlier markers. The main role for IGF-I measurement would appear to be in monitoring nutritional support in malnourished patients especially those in whom acute illness and liver disease can be excluded. It is unclear whether the levels of IGF proteins can be used to help define recovery in these patients and what should be considered the target level. A pre-morbid value could be used as the target but is unlikely to be available. Although age-related reference ranges are available, normal values vary significantly between individuals. A value toward the lower end of the reference range, for example, may be normal for one individual but not for another. Clearly when used in monitoring, IGF-I levels should be considered in clinical context along with anthropometric measurements and results of other biochemical tests. It has been suggested that relative changes in IGF-I may be more useful than absolute values [36]. Another potential use for IGF is in the prediction of relapse in AN. In this situation, body weight alone may be misleading as it reflects fluid as well as body cell mass. Investigation of this possible utility would require the IGF-I level to be studied prospectively to assess whether its decline precedes that of significant weight loss.

Refeeding syndrome (RS) can occur as a complication following commencement of nutrition support in malnourished patients, a major component of which is hypophosphataemia [105, 106]. Although guidelines make recommendations on how to recognise patients at risk of RS [25], prediction is difficult in practice and there is a need for better objective markers of risk. A recently published study on patients receiving PN examined three parameters as potential predictors of RS, namely, serum IGF-I measured before commencement of PN, the leptin level, and a “refeeding index” (RI) derived from the other two values [98]. Although RI predicted a fall in the serum phosphate level more sensitively and specifically than the other two parameters, IGF-I was a better predictor of mortality.

Serum IGF-I has significant limitations as a nutritional marker, notably that its level is likely to be reduced during the APR regardless of the nutritional state. The underlying disease must therefore be taken into account as possibly influencing its levels. Its level is also lowered in disease of the liver, the main site of IGF-I synthesis, and can be elevated or lowered in renal disease due to changes in IGFBP levels [107]. IGF-I levels have been reported to be low in patients with untreated severe hypothyroidism but increased significantly following thyroxine treatment [108]. This reflects the regulatory influence of thyroxine on IGF-I levels. Its serum level is also influenced by GH status as discussed above. Whilst its measurement may be useful in selected cases, these confounding factors mitigate against its use generally in hospital patients or in nutritional screening.

The clinical utility of measurement of free IGF-I and other proteins of the IGF system in nutritional contexts is less clear at present. If they are to be of value, the result should lead to clinical action which could potentially improve the outcome for the patient. Their utility needs to be defined by future clinical validation studies. Future research on these markers also needs to evaluate interferences and confounding factors and address the question of target values. The prognostic value of the markers needs to be determined, that is, whether they can indicate the likelihood of an adverse outcome in the event that nutritional support is withheld. Potential clinical utility for the measurement of IGF axis proteins is summarised in Table 4.

Validation studies alone are insufficient to ensure that novel tests enter the laboratory repertoire. They are unlikely to be offered widely unless recommended by clinical guidelines. Even then, logistical factors such as cost and assay availability may preclude their use. Where a test is to be of value in monitoring nutritional support, it is essential that the result is available the same day as the request so that the nutritional regimen can be modified appropriately. This necessitates the assay being sufficiently rapid and available on site. The latter may not be feasible in, for example, a district general hospital where the demand for the test is low. In the case of markers of overnutrition or insulin resistance, turnaround time for results is likely to be less critical as the target population will be mainly outpatients. The logistical problems are likely to be overcome in time as assays become cheaper and more automatable, possibly resulting in their availability as near patient tests.

## Figures and Tables

**Table 1:** The insulin-like growth factor binding proteins (IGFBPs).

IGFBP	*M*_*r*_ (kDa)	Relative affinity for IGFs	Possible functions
1	25	I = II	Inhibition of growth-promoting actions of IGFs and inhibition of insulin-like actions, integrin binding, glucose homeostasis
2	31	II > I	Stimulation of splenic growth, suppression of IGF bioactivity, cell surface binding, mitogenic for some cell types
3	29	I > II	Inhibition of IGF actions, induction of apoptosis
4	26	I = II	Inhibition or enhancement of IGF actions, inhibition of ovarian steroidogenesis, regulation of bone formation
5	28	I = II	Localises IGFs in extracellular matrix and bone, regulation of IGF bioavailability
6	23	II > I	Inhibition of actions of IGF-II

**Table 2:** Features of the ideal protein-energy marker.

Short biological half life
Exists primarily within an accessible body fluid
Limited homeostatic regulation
Constant rate of catabolism
Uninfluenced by vitamin or mineral status
Uninfluenced by pathology other than malnutrition
Measurement simple, cheap, and available locally

**Table 3:** Effect of malnutrition and obesity on serum levels of proteins of the IGF axis.

Protein	Malnutrition	References	Obesity	References
IGF-I	↓	[36, 37]	U/↑/↓	[38–45]
IGF-II	↓	[34, 46, 47]	↑	[48, 49]
Free IGF-I	↓	[50, 51]	U/↑/↓	[43–45, 48, 49, 52]
Free IGF-II	↓	[49]	↑	[49]
IGFBP-1	↑	[51]	↓	[53, 54]
IGFBP-2	↑	[34]	↓	[55, 56]
IGFBP-3	↓	[57–59]	↑	[60]

Key: ↑ increase; ↓ decrease; U unchanged.

**Table 4:** Possible clinical utility for measurement of serum IGF axis proteins.

	Reference
*IGF-I*	
Prediction of ITx patients requiring intensive nutritional intervention following transplant	[76]
Index of IF in children with SBS	[75]
Monitoring of recombinant GH treatment	[77]
Monitoring of PN and nitrogen balance	[74]
Indicator of short-term change in nutritional status	[36]
Prediction of refeeding risk and mortality	[98]
Diagnosis of acromegaly	[99]
Diagnosis of growth hormone deficiency	[99]

*IGF-II*	
Diagnosis of NICTH	[13]
Monitoring of refeeding	[34]

*IGFBP-1*	
Assessment of insulin resistance and cardiovascular risk	[96]
Marker for metabolic syndrome	[53]
Prediction of growth failure in chronic renal disease	[97]
Prediction of development of insulin resistance in children	[54]
Monitoring of life-style interventions	[95]

*IGFBP-2*	
Marker for metabolic syndrome	[55]
Diagnosis of NICTH	[100]

*IGFBP-3*	
Monitoring of nutritional status	[58]
Assessment of GH status	[101]
Monitoring of GH treatment	[101]

Key: ITx: intestinal transplant; IF: intestinal failure; SBS: short bowel syndrome; NICTH: non-islet cell tumour hypoglycaemia.
